# Feasibility of Group-Based Implementation Facilitation for Video Telemental Health

**DOI:** 10.1007/s41347-022-00295-x

**Published:** 2023-01-11

**Authors:** Anthony H. Ecker, Giselle Day, Amber B. Amspoker, Jennifer L. Bryan, Stephanie C. Day, Miryam Wassef, Kendra Weaver, Jan Lindsay

**Affiliations:** 1 Houston VA HSR&D Center for Innovations in Quality, Michael E. DeBakey VA Medical Center, Effectiveness and Safety, Houston, TX USA; 2 Education and Clinical Center (a Virtual Center), South Central Mental Illness Research, Houston, TX USA; 3 Baylor College of Medicine, Houston, TX USA; 4 Office of Mental Health & Suicide Prevention, VA Central Office, Washington, DC USA; 5 Rice University’s Baker Institute for Public Policy, Houston, TX USA

**Keywords:** Implementation facilitation, Veterans, Mental health, Telehealth, Video

## Abstract

Video telehealth experienced rapid growth throughout the COVID-19 pandemic in many healthcare sectors, including mental health. The Veterans Health Administration’s video telehealth platform, VA Video Connect, has been widely used to reach veterans who may have experienced difficulty accessing care, such as those living in rural areas or other barriers (e.g., transportation). Implementing VVC requires a multifaceted approach, including training providers on technical skills, increasing access to equipment for providers and veterans, and integrating VVC within the culture and processes of the clinic unit. Prior successful VVC implementation efforts in rural areas have focused on simultaneous one-on-one provider and leadership engagement using implementation facilitation (IF). However, given the rapid need for VVC expansion in light of limits and dangers associated with in-person care during the pandemic, our team developed group facilitation to increase the reach of VVC implementation through IF. Group facilitation combined training in technical and policy elements of VVC with IF with groups of providers from clinic units. This approach was designed to rapidly disseminate the necessary knowledge to conduct VVC combined with collaborative problem solving as a team to improve the ability of the clinical team to sustain VVC. Attendees were asked for feedback on the session through multiple choice and open-ended questions. Participants (*N* = 26) reported being highly satisfied with the training and reported a high degree of confidence in their ability to use VVC. Based on evaluation data and interview feedback, providers and clinic leaders were satisfied with group facilitation. Group facilitation may be a helpful tool in rapidly training clinical teams to implement and sustain video telemental health.

Video telehealth has experienced rapid growth throughout the COVID-19 pandemic, including for mental health (Connolly et al., 2021; Hoffman et al., 2020; Lau et al., 2020; Robinson et al., 2020; Rosen et al., 2021). The Veterans Health Administration (VHA) has been at the forefront of video telemental health (VTH) directly to patients’ homes, allowing clinicians and patients to engage through a video interface (Lindsay et al., 2017). The Department of Veterans Affairs (VA’s) VTH platform, VA Video Connect (VVC), can be accessed by different device types, including mobile technologies (i.e., smartphone or tablet) in the private location of the patient’s choosing. This technology has helped veterans across the nation receive mental health care who would not otherwise have received it (Fletcher et al., 2018; Wynn & Sherrod, 2012) and can deliver evidence-based care effectively (Morland et al., 2019). VTH has increased access to mental health care across the veteran population, especially for under-resourced groups such as individuals living in rural areas and those who prefer the convenience and comfort of receiving their healthcare at home.

Despite success expanding access to mental health care in some rural areas (Day et al., 2021), other rural settings have lagged behind other areas in implementation of VTH. The nature of adopting telehealth throughout a system may contribute to this disparity. Successful use of VTH requires technical knowledge, access to technical components (e.g., secure online video platform, cameras/audio equipment, high-speed internet), and support from the clinical context in structures that both facilitate successful telehealth integration (e.g., coding, scheduling) and cultural fit (e.g., perceived value of telecare in relation to other modalities). Implementing VTH in low-resourced areas may then require a multifaceted approach that takes into consideration these barriers and cultural components.

As a technology-based therapy modality, VTH implementation faces other barriers. Implementing VTH is multidimensional in terms of its demands on providers. Providers must learn how to use the technology itself, adapt their practice to the new modality, learn new online systems, and manage patients’ use of the technology. However, current training models may not sufficiently prepare providers, especially those in clinical contexts with less telehealth presence and infrastructure, for the multifaceted nature of telecare (Connolly et al., 2020; Rosen et al., 2021).

VHA currently offers a three-tiered, self-led, web-based VTH training for providers. The three-tiered learning choices are based on the level of provider experience with VTH (e.g., new, experienced, or expert user), with a focus on learning the basics of the technology. However, to adapt rapidly to provider, patient, and system needs, VTH platforms are updated frequently. A particular gap in training in this rapidly changing system is that communications about these frequent updates take time to permeate all levels of a national healthcare system, and frontline providers may vary in their engagement with VVC communication.

To address the needs of many providers, the VHA training is focused on providing information to reach general competence in using VVC, but it is not feasible for a training with such scope to be tailored to the myriad clinical contexts, especially during the rapidly changing context of virtual care during COVID-19. There are elements of VTH beyond skill-based learning that impact integration into clinical care. For example, providers who primarily work in the field with veterans experiencing homelessness have different VTH needs than providers transitioning patients from inpatient to outpatient care. Understandably, it is beyond the scope of broad, platform-focused training to allow providers to brainstorm solutions to potential barriers for seamless implementation of VTH in their clinic with fellow providers and administrative staff.

Implementation facilitation (IF) (Ritchie et al., 2020) is an implementation strategy with strong empirical support for sustained implementation of mental health innovations (Kirchner et al., 2014; Lindsay et al., 2019). IF is used to implement innovations in health care settings through collaborative problem solving. Facilitators work to drive successful implementation by using a range of skills to enable stakeholders (e.g., providers, administrators). These skills include collaborative problem solving, engaging with leadership, building awareness of the intervention, and other skills (Ritchie et al., 2020). IF has been successfully used to implement evidence-based practices virtually and in group/organization settings (Hartmann et al., 2021). IF has been used successfully to implement VVC in healthcare settings, including those in less-resourced rural areas (Day et al., 2021).

Central to the success of implementation of VVC in these settings was the use of both internal and external facilitators who worked one-on-one with stakeholders. External facilitators, who do not work directly for the facility, work with the internal facilitators (who are facility employees) to facilitate grass-roots efforts to increase VVC use, while maintaining connection with national efforts and leadership priorities. Although this one-on-one method enabled strong partnerships and personalized facilitation (Lindsay et al., 2015, 2019), it had several limitations. Many providers were early adopters and motivated to provide VVC. Also, reach was limited by both bandwidth of facilitators and the interest of providers (i.e., noninterested providers were not incentivized to engage). The context of top-down directives to increase use of VVC necessitated a more rapid approach with greater reach that simultaneously met the minimum threshold for providing VVC (e.g., technology training) and facilitated sustainable use of VVC in clinicians’ contexts. In light of these needs, our group built on extant IF principles (Hartmann et al., 2021; Ritchie et al., 2020) to provide facilitation in small groups formed by stakeholders from clinic units.

## Methods

The current evaluation describes the components of the group facilitation sessions and examines initial feasibility and acceptability indicators through examination of data collected in the context of ongoing quality improvement efforts. The facilitation session is conducted in a 1-h meeting led by external facilitators, attended by members of distinct clinical teams. First, group facilitation provided supplemental training in technical aspects of VVC, with the most up-to-date information, as external facilitators incorporated information from national guidelines and meetings that busy frontline providers might be unable to consistently access. This also allowed targeting training content to the type of mental health setting (e.g., outpatient pharmacotherapy and psychotherapy, inpatient, homeless outreach). Second, group facilitation allowed clinical teams to work together in one meeting to receive training in implementing VVC, problem solve together on how to best incorporate it into their setting and population, and work through their unique barriers and facilitators as a team (versus isolated single providers and champions). This also allowed team members to support each other and build a culture of shared information and resources. Attendees also brainstormed how VVC could be implemented in their particular context and how to deploy and sustain VVC as a team. Third, it allowed clinical teams to be paired with ongoing support from external and internal facilitators.

Thus far, our group has conducted 36 group facilitation sessions across nine sites from seven states. These sites were selected in partnership with VHA mental health leadership as sites that could benefit from a targeted implementation to increase use of VTH encounters. The PIVOT approach (Day et al., 2021; Lindsay et al., 2019) was used at each of the sites, an implementation package that has been used to successfully increase use of VTH in rural areas. PIVOT involves use of formative evaluation, partnership with site leadership and staff, and implementation facilitation to provide site-specific implementation support to build capacity to deliver VTH.

As a component of this ongoing quality-improvement work, attendees were invited to provide anonymous feedback through evaluation questionnaires. A total of 170 providers attended these sessions. Questionnaires evaluated attendees’ satisfaction with the training, confidence in using video telehealth, and anticipated barriers to implementing VVC. Evaluation was not required, and 26 (15% of total) attendees completed evaluation. Evaluation consisted of four multiple-choice questions and four open-ended questions. The multiple-choice questions were rated on Likert-type scales from 1 (*not at all* or *strongly disagree*) to 5 (*a great deal* or *strongly agree*). All questions are included in Table 1. Descriptive statistics of survey responses were used to evaluation quantitative feedback, and qualitative feedback was grouped into themes for each survey item.

**Table 1:** Evaluation items for group-based implementation of VA video connect

**Multiple choice items**
1. How satisfied were you with the training (1 = not at all, 5 = a great deal)
2. After the training, I feel confident in my ability to use VVC (1 = strongly disagree, 5 = strongly agree)
3. The trainers were effective at conveying knowledge (1 = strongly disagree, 5 = strongly agree)
4. The trainers kept me engaged (1 = strongly disagree, 5 = strongly agree)
**Open-ended items**
1. What did you like about the training?
2. How could the training be improved?
3. What barriers anticipate/are you experiencing in regards to using VVC
4. If you have other feedback for us, please leave comments below

## Results

### Feedback from Participants

Overall, attendees were satisfied with the training approach (*M* = 4.38, *SD* = 0.83) and rated the trainers as effective in conveying knowledge (*M* = 4.69, *SD* = 0.55) and as engaging (*M* = 4.54, *SD* = 0.76). Attendees generally rated confidence in their ability to use VVC as high (*M* = 4.38, *SD* = 0.80).

Responses to the question “What did you like about the training?” largely centered around the opportunity for interactivity and group-problem solving. Respondents noted that the ability to ask questions about their specific implementation challenges or contexts (e.g., opioid prescribing) was particularly helpful. Responses illustrative of this theme included “The trainers made the training specific to what our group was needing” and “Responsiveness to specific questions.” Another theme from respondents was the appreciation of content on the technical use of VVC and implementation suggestions. Key comments included the statement “I loved that they talked about ways to implement VVC, for example with no shows. I also loved that they talked about things to share with veterans who are apprehensive.” Other comments were “Learned new things about how to streamline my VVC visits!” and “I liked that the presenters went over everything step by step.”

Respondents also described barriers that they were experiencing or anticipate experiencing in their implementation of VVC. Such barriers included veterans’ access to equipment and sufficient high-speed internet access, patient familiarity/comfort with equipment or video calls, and scheduling concerns (e.g., ensuring patients are booked in appropriate video appointment slots). An illustrative response was “Veterans that do not have internet or if they do internet speed does not support VVC.” Responses to the remaining two open-ended questions (i.e., suggestions on training improvement and other feedback) had very few responses, and comments received largely focused on logistical elements of the training (e.g., too much/too little time spent on various content areas).

## Discussion

This initial iteration of group-based IF for building capacity to deliver VTH care enabled the PIVOT implementation team to rapidly respond to site needs for both technical and clinical implementation training. These trainings were tailored to the needs and clinical contexts of providers and staff and had a broader reach than previous one-to-one facilitation models conducted by our team (Day et al., 2021; Lindsay et al., 2017, 2019). As facilitators of the group, we noted that an unexpected opportunity emerged for group problem-solving and building a culture of innovation. That is, clinical teams were able to engage virtually with each other to determine how to best use the technology to benefit their practice and the veterans they serve. Although there is some evidence for the feasibility of this approach, more research is needed. Specifically, more systematic evaluation of group facilitation for VVC as an implementation strategy is needed.

Recent work on IF in the context of virtual facilitation identified several key practice elements, including planning in advance, real-time communication, build relationships, engaging participants, and using a virtual room (Hartmann et al., 2021). The current group facilitation used several elements of this framework, including communication and engaging participants. Future efforts would benefit from explicit incorporation of such models into the design and execution of the trainings. The group facilitation approach described reflects our team’s rapid response to site needs in the context of an ongoing multisite quality-improvement project and substantial system-wide expansion of VVC during the COVID-19 pandemic. Further studies that more rigorously evaluate implementation outcomes, such as expanded or sustained use of video telehealth, are warranted. Furthermore, it is unknown whether this approach could be used in other innovation contexts. Future work would benefit from evaluating group facilitation using other innovations or implementation targets.

The current evaluation must be considered in light of its limitations. First, the evaluation presented in this paper was initially designed for collection of feedback to improve the training in a quality improvement context. More rigorous, theoretically driven, formal evaluation is needed. This is especially the case for outcomes related to actual implementation of VVC beyond satisfaction. Furthermore, a relatively low number of those who attended the training completed the evaluation, reflecting those who were willing to share their feedback. More rigorous sampling is needed to avoid bias from such self-selection. Finally, the qualitative analysis in the current study was limited by lack of responses that precluded more rigorous forms of analysis.

Implementing health technologies is a complex task. Clinicians, staff, and other end users of these technologies have multiple considerations and demands, with varied access to resources and implementation support. The added complexity of rapid implementation of VTH during the COVID-19 pandemic precipitated the need to empower large numbers of providers and staff to implement VVC in their facilities. Combining training in technology with IF allowed training that was feasible and acceptable for interprofessional mental health staff to quickly build capacity for expanded telehealth provision.
